# Worldwide Prevalence of Dyslipidemia in Diabetes: An Umbrella Overview of the Meta-Analysis Studies

**DOI:** 10.2174/0118715303375795250521041740

**Published:** 2025-05-26

**Authors:** Zahra Hashempour, Fataneh Esmaeili, Ozra Tabatabaei-Malazy, Asieh Mosallanejad, Ghodratollah Panahi

**Affiliations:** 1 School of Medicine, Shiraz University of Medical Sciences, Shiraz, Iran;; 2 Department of Clinical Biochemistry, School of Medicine, Tehran University of Medical Sciences, Tehran, Iran;; 3 Non-communicable Diseases Research Center, Endocrinology and Metabolism Population Sciences Institute, Tehran University of Medical Sciences, Tehran, Iran;; 4 Endocrinology and Metabolism Research Center, Endocrinology and Metabolism Clinical Sciences Institute, Tehran University of Medical Sciences, Tehran, Iran;; 5 Imam Hosein Medical Center, Shahid Beheshti University of Medical Sciences, Tehran, Iran

**Keywords:** Dyslipidemia, diabetes mellitus, lipoprotein cholesterol, vascular complications, meta-analysis, umbrella review

## Abstract

**Background:**

Dyslipidemia, a modifiable risk factor for Cardiovascular Diseases (CVDs), is prevalent among individuals with Diabetes Mellitus (DM). The coexistence of DM and dyslipidemia exacerbates the burden of CVDs. Given the variability in findings across systematic reviews, this umbrella review aims to assess the prevalence of dyslipidemia among diabetic patients critically.

**Methods:**

A systematic search was performed across PubMed, Scopus, Web of Science, and Embase databases to identify meta-analyses addressing the prevalence of dyslipidemia in patients with DM. Studies were selected in accordance with the Preferred Reporting Items for Systematic Reviews and Meta-Analyses (PRISMA) guidelines. Meta-analyses that provided data on the prevalence or mean difference of lipid profile components in diabetic patients were included. To evaluate study quality, the Assessment of Multiple Systematic Reviews-2 (AMSTAR-2) and Grading of Recommendations Assessment, Development, and Evaluation (GRADE) frameworks were applied, ensuring the reliability and consistency of the findings.

**Results:**

Eleven meta-analyses with a total sample size ranging from 433 to 354,088 participants were included. The prevalence of overall dyslipidemia varied between 60% and 65.68%. Specific lipid abnormalities were also prevalent: high total cholesterol (34.7–38.6%), elevated triglycerides (43–52.7%), high low-density lipoprotein cholesterol (34.4-41%), and low high-density lipoprotein cholesterol (43.4–50%). Gender differences were insignificant, with a higher prevalence of dyslipidemia among women compared to men, particularly after menopause (19% *vs.* 18%).

**Conclusion:**

Dyslipidemia is highly prevalent among diabetic patients, with significant gender-specific patterns, particularly affecting postmenopausal women. These findings highlight the importance of early screening and targeted management of lipid abnormalities in DM patients to reduce the risk of vascular complications. Furthermore, the quality assessment indicated that most included studies were of low or very low quality, highlighting the need for more robust research in this field.

## 1 INTRODUCTION

The global prevalence and incidence of dyslipidemia and diabetes have been rising, with a dominant increase in Type 2 Diabetes (T2DM) [1, 2]. The term dyslipidemia refers to a cluster of metabolically related abnormalities that affect plasma lipids and lipoproteins, including the sole increase in Low-Density Lipoprotein Cholesterol (LDL-C), Triglyceride (TG), and Total Cholesterol (TC), or decrease in High-Density Lipoprotein Cholesterol (HDL-C) levels, or a combination of all the above changes [3]. The prevalence of dyslipidemia among T2DM patients is alarmingly high, with rates ranging from 64-87.5% [4-6]. The higher rate of dyslipidemia in DM could be related to insulin resistance or key enzyme deficiency in lipid metabolism pathways [7]. Moreover, dyslipidemia is one of the main risk factors for vascular complications in diabetic patients [8].

Both DM and dyslipidemia are modifiable risk factors that can independently enhance the risk of Cardiovascular Diseases (CVDs). Population-based studies indicate that individuals with T2DM face a significantly higher risk of developing CVDs compared to those without the condition [9, 10], with more than half of them dying from CVDs [11, 12]. Additionally, the higher rates of dyslipidemia in T2DM lead to a higher risk of CVD and death [3, 11]. Therefore, early screening, timely diagnosis, and effective intervention for dyslipidemia can decrease the risk of CVDs [3, 11, 13].

Despite the importance of dyslipidemia and its complications in DM, systematic reviews regarding the prevalence of dyslipidemia among these patients are scarce and inconsistent. Moreover, no prior study has conducted an umbrella overview of the available meta-analytic evidence. Therefore, we aimed to conduct an umbrella review of the meta-analysis studies regarding the prevalence of dyslipidemia in patients with DM to provide a broader, higher-level assessment of the patterns, inconsistencies, and limitations across meta-analysis studies.

## 2 METHODS

This study was conducted in accordance with the Preferred Reporting Items for Systematic Reviews and Meta-Analysis (PRISMA) guidelines [14].

### 2.1 Search Strategy

A comprehensive search was conducted across PubMed, Scopus, Web of Science, and Embase web databases to identify pertinent meta-analyses exploring the prevalence of dyslipidemia in patients with diabetes up to 1 September 2024. Hand-searching was conducted to identify additional relevant studies. The PECO (Participant, Exposure, Comparison/Control, Outcome) framework was employed to systematically review the literature on the prevalence of dyslipidemia in the DM population and lipid profile components, including cholesterol, triglycerides, HDL-C, and LDL-C. Only English language publications were included in the study. To reduce the likelihood of overlooking relevant studies, the primary data collection strategy combined MeSH terms with keywords (Table **S1**).

### 2.2 Inclusion and Exclusion Criteria

Research studies published in English were eligible for inclusion if they fulfilled the following criteria: 1) Systematic reviews and meta-analyses of observational studies (cross-sectional, cohort, case-control), 2) Adult participants (age ≥ 18 years) with DM of any gender, and 3) Reported the prevalence, or mean difference of total or each component of the lipid profile, including TC, TG, LDL-C, and HDL-C in individuals with DM.

The exclusion criteria comprised animal studies, gestational diabetes mellitus or pediatric population, meta-analyses of clinical trials, case reports, editorials, narrative reviews, and systematic review studies lacking meta-analysis.

### 2.3 Extraction of the Data

Two researchers (ZH and FE) independently screened the titles, abstracts, and keywords of all studies retrieved from online database searches for possible inclusion in the current study. All eligible references from the initial screening were thoroughly reviewed in full text to ensure inclusion. Two authors (ZH and FE) extracted the relevant data from the included articles independently. Disagreements were resolved through discussion and consensus supported by consultation with the third author (OTM). All in all, two processes were used: 1) Disagreements between reviewers during the title and abstract screening were resolved through consensus discussions, and 2) In cases where consensus could not be reached, a third senior reviewer was consulted to resolve conflicts. The extracted information encompassed title, leading author, publication year, population characteristics (total number, age, sex, underlying disorder, and lipid profile), the number and design of the studies, results of meta-analyses, and subgroup analyses. In cases where full-text articles were unavailable or certain information was incomplete, corresponding authors were contacted for assistance.

### 2.4 Quality Assessment

The quality of the included systematic reviews and meta-analyses was evaluated using the “A Measurement Tool to Assess Multiple Systematic Reviews–2” (AMSTAR2) [15]. The findings of this evaluation are outlined in Table **S2**. Furthermore, the Grading of Recommendations Assessment, Development, and Evaluation (GRADE) framework was applied to assess the quality of evidence, and the results are presented in Table **1** [16].

## 3 RESULTS

### 3.1 Characteristics of the Studies

Overall, 11 papers were included in the study [6, 17-26]. The search process, following the PRISMA flowchart, is illustrated in Fig. (**1**). These studies were subsequently categorized into two distinct groups based on the metric assessments: 1) Standardized or weighted mean difference (SMD, WMD) and 2) Prevalence. Detailed information on the included studies is presented in Table **1**.

All selected studies were meta-analyses of observational studies, encompassed both genders, and examined dyslipidemia or its components in patients with diabetes or diabetic complications. The sample size of the population ranged from 433 to 354,088 participants, aged 11 to 90 years. Participants were suffering from various underlying conditions, including Type 1 Diabetes Mellitus (T1DM), T2DM, Diabetic Foot (DF), Diabetic Retinopathy (DR), and Metabolic Syndrome (MetS). The quality assessment was performed for most of the included meta-analyses, with further details about the included studies outlined below.

### 3.2 Pooled Mean Difference of Lipid Profiles

Overall, in six observational studies, the SMD, WMD, or MD of the lipid profiles were reported [21-26]. These figures for SMD or WMD of high TC, high TG, high LDL-C, and low HDL-C mg/dl ranged from -0.77 to 17.79 in DF patients, 2.94 to 82.3 in DR, 2.53 to 17.02 in DR, and -34.42 to 9.67 in DF, respectively [21, 22, 25, 26].

### 3.3 Prevalence of Lipid Profiles

A total of five papers were incorporated in the assessment of the prevalence of dyslipidemia in diabetes [6, 17-20]. Out of these two studies reported to total dyslipidemia: Azagew *et al*., (65.68%) in Ethiopia, and Khalil *et al*., (60%) in Middle East countries [17, 18]. Overall, the prevalence of high TC, TG, LDL-C, and low HDL-C were 34.7-38.6%, 43-52.7%, 34.4-41%, and 43.4-50%, respectively [6, 17-19]. One study that assessed the prevalence of concomitant hypertriglyceridemia and central obesity estimated the prevalence as 19, 18, and 18 percent, respectively, in females, males, and both genders [20].

### 3.4 Quality Assessment

The quality of the included studies was assessed using the AMSTAR-2 and GRADE frameworks. Among the five studies reporting the prevalence of dyslipidemia, one was of medium quality, while the remaining four were rated as low quality. Similarly, six studies focusing on mean differences in lipid profiles showed varied quality: two moderate, two low, and two very low. Items seven and ten accounted for most of the limitations, as they required a list of excluded studies with justifications and disclosure of funding sources for each included study, respectively. The presence of these variations among the limited number of studies highlights a critical gap in the literature on this prevalent and clinically significant topic. Since this study is an umbrella overview of the meta-analyses, the quality of the summarized evidence inherently depends on the strength of the included studies. Therefore, future systematic reviews in this field should prioritize higher methodological rigor.

## 4 DISCUSSION

This study revealed that the total prevalence of dyslipidemia in patients with DM varies from 60% to 65.68% worldwide. The highest prevalence was attributed to LDL-C (52.7%), followed by TG levels (51.8%). According to the meta-analyses of the case-control studies, the highest mean difference was reported for hypertriglyceridemia in diabetic patients compared to the control group. Also, among the evaluated lipid components in each study, increased TG levels had the highest mean difference.

There is a close interrelationship between lipid and glucose metabolism [27]. Although serum lipid levels are elevated with the degree of hyperglycemia, hyperinsulinemia compensated by insulin resistance correlates as well [28]. Research shows a higher prevalence of dyslipidemia in prediabetic and diabetic individuals, compared to non-diabetics [29]. Moreover, studies confirm that hypertriglyceridemia is the most common lipid abnormality among diabetic patients, which is consistent with the highest mean difference of TG reported in the included research of our review [17, 21, 22, 24, 28]. However, the prevalence of dyslipidemia can vary depending on the type of diabetes. In a retrospective analysis of diabetic adolescents and young adults, Kim *et al*. reported higher rates of uncontrolled LDL-C in T2DM than in T1DM individuals [30]. Three of the included studies in this umbrella systematic review involved diabetic populations of both types, but did not differentiate the results accordingly [17, 23, 24]. Further investigation is warranted to determine the exact distribution of dyslipidemia among these populations.

According to a study by Ren *et al*., the overall prevalence of Hypertriglyceridemic Waist (HTGW) was mildly higher in women than in men (19% *vs.* 18%), indicating that dyslipidemia is insignificantly more common among women in the context of diabetes. This study shows that while both genders with HTGW are at increased risk of T2DM, women show a higher prevalence and a greater associated risk, likely due to hormonal and metabolic factors [20]. Similarly, high-risk TG levels were slightly more prevalent in diabetic women than in men in other studies [31]. A recent cross-sectional study showed that while younger women with T2DM had more favorable lipid profiles compared to men, they had higher LDL-C levels than men when they were older than 50 years [32]. Additionally, Paquet *et al*. declared that women with T2DM have higher LDL-C levels than men, regardless of the treatment or cardiovascular risk level [33]. These findings highlight the need for tailored lipid profile management strategies to address gender-specific cardiovascular risks in diabetic dyslipidemia.

Multiple risk factors affect the prevalence of dyslipidemia in the diabetic population. Even with normal glucose levels, smoking is associated with an increased prevalence of dyslipidemia [29]. Nicotine causes a series of biochemical, hormonal, and metabolic disruptions after entering the bloodstream. These effects seem to be substantially more severe in individuals with diabetes [34, 35]. Kar *et al*. evaluated the distribution of dyslipidemia components in the diabetic population, and compared the results between smokers and non-smokers. This meta-analysis revealed that non-smokers had more favorable lipid profiles, with higher levels of HDL-C and lower levels of LDL-C compared to smokers. Specifically, the mean difference in HDL-C between smokers and non-smokers was 0.12 mmol/L (*p* < 0.001), and in LDL-C, the difference was -0.11 mmol/L (*p* = 0.03) [23]. These differences suggest that smoking exacerbates dyslipidemia in diabetic patients, contributing to a more atherogenic lipid profile, which could heighten the risk of cardiovascular complications. It is crucial for clinicians to address the importance of smoking cessation as a critical part of dyslipidemia management.

Serum lipids are involved in the occurrence and exacerbation of vascular complications of diabetes. In a recent meta-analysis by Li *et al*., elevated TC, TG, and LDL-C levels were associated with an increased risk of DR, underscoring the detrimental effects of dyslipidemia on the microvascular complications of diabetes [26]. Similarly, LDL-C may be considered a risk factor for the occurrence of diabetic polyneuropathy [25]. Nevertheless, in a previous meta-analysis, no significant difference in TC, TG, and HDL-C levels was observed between diabetic patients with and without retinopathy. However, slightly elevated LDL-C was observed in patients with DR [24]. Furthermore, Pei *et al*. demonstrated that reduced HDL-C levels are significantly linked to the development of DF, a macrovascular complication, though other lipid parameters (TC, TG, LDL-C) did not exhibit strong associations. According to this study, while lowering LDL-C is a priority for cardiovascular risk management, increasing HDL-C levels should also be a focus in the prevention of diabetic foot complications [21]. This is crucial since DF is considered a leading cause of non-traumatic amputations, and DR accounts for a major cause of vision loss in diabetic patients [36, 37]. While the studies examine different complications, they collectively emphasize the need for effective lipid management and potentially aggressive lipid-lowering strategies in patients with DM.

This umbrella review study presents significant strengths. This study provides a comprehensive approach to overview data from the meta-analyses on the prevalence of dyslipidemia among DM patients. While several systematic reviews and meta-analyses have explored the prevalence of dyslipidemia in diabetes, to researchers’ knowledge, this study is the first umbrella overview of these meta-analyses. Unlike traditional systematic reviews, this approach provides a higher-level synthesis, identifying broader trends, methodological limitations, and research gaps, while enhancing the evaluation of consistency, bias, and future research needs.

However, this review has some limitations that should be acknowledged. First, the included studies were limited to those published in English, which may have resulted in the exclusion of relevant research published in other languages. Second, the included meta-analyses were primarily observational, and the inherent biases associated with observational studies, such as confounding factors and selection bias, may have influenced the overall findings. Finally, we could not distinguish the prevalence of dyslipidemia in the studies that included both type 1 and type 2 diabetes, as the studies did not provide separate prevalence estimates for each population. Future meta-analyses should focus on distinguishing dyslipidemia prevalence in Type 1 and Type 2 diabetes patients to allow for more targeted clinical recommendations.

## CONCLUSION

This umbrella systematic review aimed to identify the findings and contents of meta-analyses related to the dyslipidemia prevalence in patients with DM. This review underscores the high prevalence of dyslipidemia among diabetic individuals, and its contribution to the progression of both microvascular and macrovascular complications. These results provide clinically valuable evidence regarding the impact of dyslipidemia and how its effective management remains essential for reducing the burden of vascular complications in diabetic populations. Clinicians should incorporate routine lipid screening in diabetes management protocols, particularly high-risk subgroups, such as postmenopausal women and smokers. Future research should prioritize well-designed studies to address inconsistencies related to testing methods and the definition of dyslipidemia, ensuring more precise and actionable clinical insights.

## Figures and Tables

**Fig. (1):**
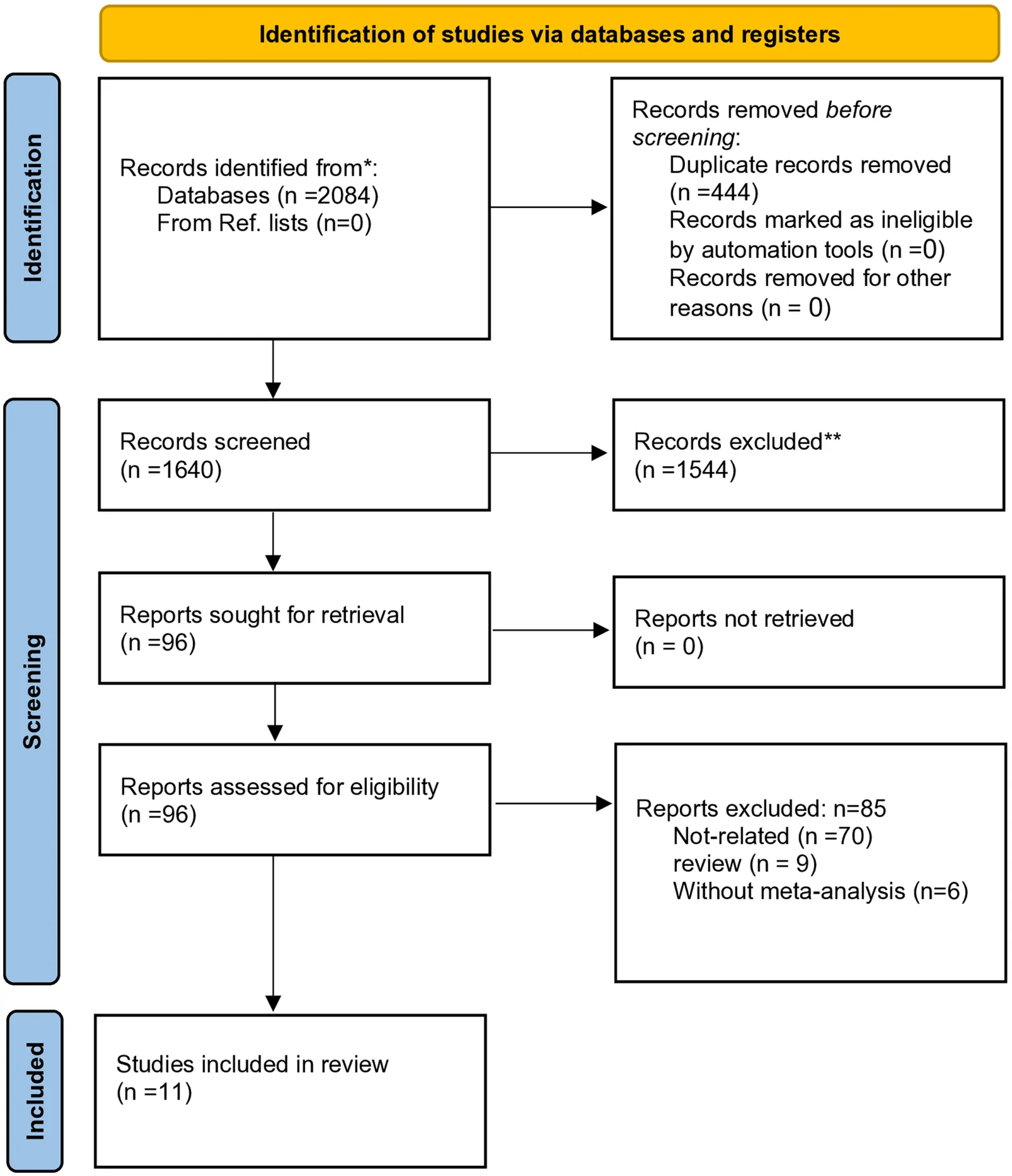
**-** PRISMA flowchart of the study review process.

**Table 1:** Characteristics of the included studies.

Sr. No.	Authors (year)	Design/ Country	Study Population	Dyslipidemic/ non-dyslipidemic (N)	Sex (N)	Mean Age (y)	Mean DM duration (year)	Included Studies (N)	Abnormal Lipid profile	Quality Assessment (yes/no)	Effects Model	MA metric	MA Outcomes			Heterogeneity		GRADE level
													Estimates	95% CI	*p* Value	I^2^ (%)	*p* Value	
Prevalence																			
**1**	Azagew (2024)^17^	Cross-sectional, case-control/ Ethiopia	T1DM, T2DM	NR	B: 3662	≥18	style="background-color:#cdcdcd;">NR	14 (13 cross-sectional, 1 case-control)	All	Yes	Random	Prevalence	65.68	57.45, 73.92	NR	97	˂0.0001	OO 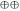 Low
									TG				51.8	45.1, 58.6		94.3	0.09	
									HDL-C				44.2	32.8, 55.7		98.2	0.85	
									LDL-C				34.4	22.3, 46.6		97.5	0.11	
									TC				34.7	23.3, 46		97.3	0.39	
**2**	Ekpor (2024) ^6^	Cross-sectional/ Africa	T2DM	NR	M: 8454 F: 11580 B: 20034	49.6 - 63.3	4-12	60	All	Yes	------	------	-------	-------	------	-------	-------	OO 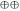 Low
					10488			31	TC		Random	Prevalence	38.6	34.1, 43.3	0.22	95	˂0.01	
					5802			21	LDL-C				52.7	44.2, 61.1	0.45	97	˂0.01	
					10594			35	HDL-C				43.5	37.1, 50	0.40	97	˂0.01	
					17216			51	TG				37.4	32.2, 42.9	˂0.01	98	˂0.0001	
**3**	Khalil (2024) ^18^	Clinical trials and observational/ Middle East & North Africa (MENA) region	T2DM	NR	-----	------	NR	------	All	Yes	Random	Prevalence	60	51, 68	˂0.01	99	NR	OO 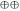 Low
									TC				37	33, 42		99		
									TG				43	38, 48		98		
									LDL-C				41	33, 49		99		
									HDL-C				50	42, 57		99		
**4**	Musilanga (2024) ^19^	Cross-sectional/ sub-Saharan Africa	T2DM	NR	Both: 4638		NR	Total:15 NCEP-ATP III 2004: 9 IDF: 6	HDL-C	Yes	Random	Prevalence	NCEP-ATP III: 43.3 IDF: 49.9	33.5, 53.2 37.3, 62.6	NR	NR	NR	OO 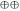 Low
									TG				NCEP-ATP III 2004: 48 IDF: 49.2	35.2, 60.7 34.1, 64.4				
**5**	Ren (2016) ^20^	Case-control, cross-sectional/ China	T2DM	NR	M: 43107 F: 39828 No gender distinction: 10259	11-90	NR	25	Hypertriglyceridemic waist	Yes	Random	Prevalence	F:19 M: 18 B:18	13, 24 13, 23 13, 23	NR	99.5 99.5 99.7	<0.0001	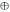 O 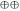 Moderate
Mean Differences																			
**6**	Pei (2014) ^21^	Case-control/ China	T2DM (Effect of lipids on Diabetic foot)	NR	M: 224 F: 209 B: 433	61.19 (11.00)	2-24	4	TG TC LDL-C HDL-C	Yes	Random	SMD (mmol/L)	TG: 0.08	-0.11 , 0.26	0.410	40	NR	OO 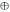 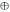 Low
													TC: -0.02	-0.22 , 0.18	0.830	40	NR	
													LDL-C: -0.10	-0.28 , 0.09	0.312	40	NR	
													HDL-C: -0.25	-0.53 , 0.03	0.085	52.5	0.097	
											Fixed		HDL-C: -0.20	-0.39 , -0.01	0.035			
**7**	Zhu (2015) ^22^	Case-control /China	T2DM	NR	B: 4010	40.7-69.5	NR	15	TG, TC, HDL-C, LDL-C	NR	Random	SMD (mmol/L)	TG: 0.93 TC: 0.46 HDL-C: -0.89 LDL-C: 0.44	0.78,1.09 0.21,0.71 -1.18 , -0.60 0.11,0.77	NR	48.8 86.5 88.1 90.8	0.040 0.000 0.000 0.000	OO 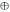 O Very Low
**8**	Kar (2016) ^23^	Cross-sectional/ World	T1&2DM (smokers *vs.* non-smokers)	NR	B: 34124	>16, <22	NR	6	HDL-C	Yes	Random	MD (mmol/L)	0.12	0.08, 0.15	<0.00001	42	0.13	OO 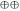 Low
									LDL-C				-0.11	-0.21, -0.01	0.03	57	0.04	
**9**	Zhou (2018) ^24^	Case-control/ China	T2DM (Relationship between lipid profile and DR)	NR	B: 4366	Combined: 60.12 (17.09)	NR	7	TG: 7	Yes	Random	MD (mg/dL)	9.18	-4.14 , 22.49	0.18	65	0.009	OO 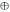 O Very Low
											Fixed		3.22	2.08, 8.52	0.23			
									TC: 6		Random		3.77	-2.45 , 9.98	0.24	41	0.13	
											Fixed		2.61	-1.52 , 6.74	0.22			
									HDL-C: 5		Random		-1.14	-2.43 , 0.15	0.08	0	0.84	
									LDL-C: 4		Random		3.74	0.13,7.35	0.04	20	0.29	
											Fixed		3.55	0.45, 6.66	0.02	20	0.29	
**10**	Naqvi (2019) ^25^	All types of observational studies/ World	T2DM	NR	M: 168,095 F: 185,993 B: 354,088	60.11 ± 10.00	5-15	36	LDL-C	Yes	Random	SMD (mmol/L)	0.077	0.025-0.129	0.004	88.1	<0.001	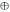 O 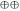 Moderate
		Case-control						11					0.031	-0.046 , 0.108	0.433			
		Cohort						3					0.160	-0.048 , 0.367	0.132			
		Cross-sectional						22					0.073	0.012, 0.135	0.019			
**11**	Li (2023) ^26^	Cohort/ World	T2DM and retinopathy	NR	M: 3882 F:3577 B:7459	44-73	4-11	Total: 13 TC: 13	TC, TG, LDL-C, HDL-C	Yes	Fixed	WMD (mg/dl)	TC: 2.94	1.32, 4.56	< 0.001	11	0.335	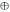 O 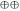 Moderate
								TG:11					TG: 8.13	5.59, 10.66	< 0.001	39	0.089	
								LDL-C: 7					LDL-C: 2.53	1.02,4.04	0.001	0	0.668	
								HDL-C: 9			Random		HDL-C: 0.27	-0.91, 1.45	0.656	59.1	0.012	

**Abbreviations:** NR: not reported, MA: meta-analysis, T2DM: type 2 diabetes mellitus, COTs: cross over trials, RR: relative risk, OR: odds ratio, HR: hazard ratio.

## STANDARDS OF REPORTING

PRISMA guidelines were followed.

## SUPPLEMENTARY MATERIALS

Supplementary tables and PRISMA checklist is available on the publisher’s website along with the published article.
**Table S1-** Search strategy in PubMed.
**Table S2-** Results of critical appraisal of the included meta-analysis studies by AMSTAR2.
